# The mitogen and stress-activated protein kinase 1 regulates the rapid epigenetic tagging of dorsal horn neurons and nocifensive behaviour

**DOI:** 10.1097/j.pain.0000000000000679

**Published:** 2016-08-02

**Authors:** Keri K. Tochiki, Maria Maiarú, Caspar Norris, Stephen P. Hunt, Sandrine M. Géranton

**Affiliations:** Department of Cell and Developmental Biology, University College London, London, United Kingdom

**Keywords:** Histone H3, p-H3S10, MSK1, ERK1/2, Formalin

## Abstract

Phosphorylation of histone H3 at serine 10 (p-H3S10) is a marker of active gene transcription. Using cognitive models of neural plasticity, p-H3S10 was shown to be downstream of extracellular signal-regulated kinase (ERK) signalling in the hippocampus. In this study, we show that nociceptive signalling after peripheral formalin injection increased p-H3S10 expression in the ipsilateral dorsal horn. This increase was maximal 30 minutes after formalin injection and occurred mainly within p-ERK-positive neurons. Spinal p-H3S10-enhanced expression was also observed in neurokinin 1 receptor (NK1R), c-Fos, and Zif268 positive neurons and was inhibited by ablation of serotonergic descending controls. The mitogen and stress-activated protein kinase 1 (MSK1) is downstream of ERK and can induce p-H3S10. We found that, after formalin injection, most phospho-MSK1 (p-MSK1)-positive cells (87% ± 3%) expressed p-ERK and the majority of p-H3S10-positive cells (85% ± 5%) expressed p-MSK1. Inhibition of ERK activity with the MEK inhibitor SL327 reduced formalin-induced p-ERK, p-MSK1, and p-H3S10, demonstrating that spinal p-MSK1 and p-H3S10 were at least partly downstream of ERK signalling. Crucially, pharmacological blockade of spinal MSK1 activity with the novel MSK1 inhibitor SB727651A inhibited formalin-induced spinal p-H3S10 and nocifensive behaviour. These findings are the first to establish the involvement of p-H3S10 and its main kinase, MSK1, in ERK regulation of nociception. Given the general importance of ERK signalling in pain processing, our results suggest that p-H3S10 could play a role in the response to injury.

## Abstract 2

Supplemental Digital Content is Available in the Text.

Signalling events downstream of extracellular signal-regulated kinase activation lead to the rapid epigenetic tagging of dorsal horn neurons by p-H3S10 and regulate the development of pain states.

## 1. Introduction

It is well established that activation of the extracellular signal-regulated kinase (ERK) regulates synaptic plasticity in neural networks throughout the central nervous system.^2,14,33^ Peripheral injury activates nociceptors and promotes the rapid expression of phospho-ERK (p-ERK) in superficial dorsal horn neurons, leading to central sensitization and lowered nociceptive thresholds.^23^ Thus, inhibition of p-ERK results in the reduction in the sensitised state.^32^ How ERK mediates these changes is largely unknown, but there is evidence to suggest that potassium channels, specifically Kv4.2, synaptic vesicle trafficking, translation factors, and cytoskeletal proteins are modulated by ERK.^28,30,33^ Moreover, changes in gene expression are known to underlie the plasticity that contributes to the full manifestation of pain states^37^ and recent data raised the possibility that ERK could control gene expression through epigenetic histone modifications.^16^

Epigenetic modifications of histone proteins or DNA are now known to play an important role in the dynamic regulation of gene expression and neural plasticity.^5,56^ Transcriptionally permissive histone modifications on histone H3, particularly phosphorylation at serine 10 (p-H3S10) and also acetylation at lysine 14 (AcH3K14), or a combination of these 2 marks, are upregulated in the hippocampus in association with consolidation of fear memory and on exposure to environmental novelty.^12,26,39^ The signalling pathways that initiate these changes have not been fully identified. However, it has been shown that ERK activity regulates memory formation in hippocampal neurons through the mitogen and stress-activated protein kinase 1 (MSK1) and histone phosphorylation at serine 10 (p-H3S10).^13,14^ Moreover, ERK/MAPK signalling is required for the synaptic activity-induced, NMDA-mediated, nuclear infolding seen in hippocampal neurons, a process that correlates with phospho-MSK1 (p-MSK1) and p-H3S10 expression levels.^54^ Finally, genetic inactivation of MSK1 reduces the phosphorylation of histone H3 at serine 10 at the FosB promotor in the striatum.^22^

Although epigenetics studies have elucidated a role for spinal histone acetylation and histone-acetylating enzymes in the regulation of acute and long-term pain behaviours,^3,11,20,21,25,51^ injury-induced histone phosphorylation in the spinal cord has not yet been investigated. Given the importance of ERK signalling during the early phase of pain processing after injury,^32^ the potential role of the kinase in the phosphorylation of histone H3,^16^ and the role of p-H3S10 in neural plasticity, we have investigated the possibility of rapid epigenetic p-H3S10 tagging of dorsal horn neurons through ERK signalling after noxious stimulation, using molecular and behavioural techniques, combined with pharmacological inhibition of signalling pathways.

## 2. Methods

All procedures were licensed under the Animals (Scientific Procedures) Act 1986. Experiments were performed using adult male Sprague-Dawley rats at 200 to 230 g at the beginning of the experiments and bred by the UCL Biological Services Unit. For experiments in which animals required intrathecal (i.t.) surgery, all animals weighed between 170 and 185 g on the day of the i.t. surgery. Animals were housed 4 maximum to a cage, with a 12-hour light–dark cycle (lights on beginning at 08:00) and access to food and water provided ad libitum.

### 2.1. Models of acute pain

Acute inflammation was induced with an intraplantar hind paw injection of formalin (33.3 μmol or 1 mg in 50 μL, 2%) or capsaicin (25 μL, 1%) under isoflurane anaesthesia (5% for induction and 2.5% for maintenance under nose cone combined with 100% O_2_, 2 L/min) using a 1-mL syringe and disposable 30-G ½-inch needle. After anaesthesia, animals were returned to a temperature-controlled recovery box until regaining consciousness. Sham animals were subjected to the same anaesthesia that was used for the injected animals but received no injection. Any injection would indeed trigger inflammation and therefore activate nociceptive pathways in the superficial dorsal horn, only to a different degree.

To allow for behavioural observation after formalin, a nonanaesthetised injection was required. Because of Home Office licence limitations, formalin injections performed without anaesthesia were delivered in 20 μL (33.3 μmol or 1 mg; 5%), during which the animal was lightly restrained in a leather glove. The survival times after treatment ranged from 5 minutes to 4 hours depending on the experiment.

### 2.2. Behaviour

Formalin-induced behaviours were recorded with a Panasonic SDR-H100 video camera for 60 minutes after formalin injection with animals placed in an 11- × 17-cm perspex box. Behaviour was scored and quantified as the length of time spent licking and flinching, or the number of total flinches in 5-minute bins across the full 60 minutes after injection. All scoring was done with the experimenter blind to treatment.

### 2.3. Drugs

Desipramine (Sigma D3900; i.p. 25 mg/kg per 0.9% saline), a norepinephrine reuptake inhibitor, was administered 1 hour before 5,7-dihydroxytryptamine creatinine sulphate (5,7-DHT) to prevent toxicity to noradrenergic neurons. 5,7-DHT (Sigma 37970; i.t. 60 μg in 10 μL/0.9% saline) was used to ablate descending serotonergic fibres from the rostral ventromedial medulla (RVM) and administered 7 days before formalin stimulation. SL327 (Tocris 1969; i.p. 50 mg/kg per DMSO; Tocris Bioscience, Bristol, United Kingdom) and SB747651A (Tocris 4630; i.t. 10 μM in 10 μL or 1 μM in 10 μM per 0.0086% ethanol per 0.9% saline) were delivered 30 minutes before formalin stimulation.

### 2.4. Intrathecal surgery

Surgery was performed under isoflurane anaesthesia (5% for induction and 2.5% for maintenance under nose cone combined with 100% O_2_, 2 L/min). The head of the animal was shaved, and the animal was secured in a stereotaxic frame. After sterilisation of the shaved area, an incision was made using a #22-blade from the top of the head to the base of the skull/first vertebra. The muscle was parted to reveal the atlanto-occipital membrane, which was carefully pierced and a small opening was cleared to allow insertion of a polyethene tube (diameter 0.28 mm) attached to a 50 μL Hamilton syringe containing either drug or vehicle. The tubing was carefully inserted into the subarachnoid space and guided to a predetermined location identifying the L4/L5 spinal cord region and 10 μL of solution delivered. The tube was inserted without resistance, and care was taken not to damage the spinal cord or dorsal roots. Once the tube was removed, the overlying muscle was sutured using 5-0 Mersilk, and the wound was subsequently closed with three 11- × 2-mm suture clips. The animal was then placed into a temperature-controlled recovery box and monitored closely for signs of locomotor impairment or distress once awake. Animals receiving 5,7-DHT were also monitored for any weight loss after surgery for 6 days.

### 2.5. Perfusion and tissue collection

After formalin stimulation, animals were deeply anaesthetised first with isoflurane and subsequently with pentobarbital (Euthatal; i.p 0.8 mL/animal). After breathing stopped and the animal failed to exhibit eyelid reflex, a transcardial perfusion was performed first with heparinised saline (5000 IU/L 0.9% saline) followed by 4% paraformaldehyde (PFA)/0.1M phosphate buffer (PB) for 7 minutes, using a pump and 18-gauge cannula delivering the fixative at 30 mL/min. The PFA solution contained 0.2% (wt/vol) sodium fluoride (NaF), a phosphatase inhibitor. The spinal cord was extracted and postfixed for 2.5 to 3 hours in the same PFA solution and subsequently stored at 4°C in 30% sucrose/0.1 M PB/0.02% (wt/vol) azide for cryoprotection for at least 48 hours before further processing.

### 2.6. Immunohistochemistry

Antibodies were detected with either fluorescent (Alexa Fluor conjugate or tyramide signal amplification) or chromogenic (3,3′ diaminobenzidine [DAB]) methods. The L4 to L6 region of the spinal cord was isolated for sectioning on a Leica SMR2000R freezing microtome. Serial 40-μm coronal sections were taken in 6 sets and stored in 5% sucrose/0.1 M PB/0.02% (wt/vol) azide until immunohistochemistry. All immunohistochemistry was done free floating in volumes of 1 mL with gentle rocking agitation unless otherwise stated. Three 10-minute washes in 0.1 M PB were performed after incubation with all solutions except after incubation with blocking solution. Washes were done in excess of 1 mL.

In some cases, an antigen retrieval step was required (for p-H3S10 when using a direct protocol, see below). In this case, sections were acid-pretreated with 2 N hydrochloric acid (HCl) for 30 minutes followed directly by a neutralisation step of 0.1 M boric acid for 10 minutes before serum block. Sections were blocked for 1 hour in 3% normal goat or horse serum (Vector Laboratories, Peterborough, United Kingdom) depending on the host of the secondary antibody, and 0.3% triton X-100 in 0.1 M PB to minimise nonspecific binding of secondary antibodies. For chromogenic staining, 2% H_2_O_2_ was added to the blocking solution to quench endogenous peroxidase activity and minimise background. After removal of the blocking solution, primary antibody was added overnight (O/N) at room temperature (RT) or over 3 days (5-HT, NK1, p-H3S10, and p-MSK1) at 4°C in Triton-Tris-buffered saline. Where a double stain was completed with 2 antibodies of the same host, a negative control eliminating the second primary confirmed no cross reactivity of secondary antibodies. 5-HT (Chemicon/Merk Millipore, Darmstadt, Germany MAB352; 1:75) was detected using Biotin Amplification as previously described.^24^ Antibodies detected using tyramide signal amplification as previously described^51^ were used as follows: c-Fos (Calbiochem, San Diego, CA, PC38, 1:20,000), NK1R (Merk Millipore, Darmstadt, Germany AB5060, 1:20,000), nNOS (Cell signalling, Danvers, MA, 4231S, 1:1000), p-ERK (Thr202/Tyr204) (Cell signalling 9101), p-H3S10 (Abcam, Cambridge, United Kingdom, ab14955, 1:10,000), p-MSK1 (Thr581) (Cell signalling 9595, 1:500), and Zif268 (Egr-1) (Santa Cruz Biotechnology, Dallas, TX sc-189, 1:10,000). When using a conventional DAB protocol for detection, antibodies were used at the following concentration: c-Fos, 1:10,000; p-ERK (Thr202/Tyr204), 1:500; and p-H3S10 1:5000. Antibodies detected using Alexa Fluor conjugates were NeuN (Chemicon MAB377, 1:1000); p-ERK (Thr202/Tyr204) (1:500); p-H3S10 (1:500, O/N; HCl pretreatment required), Iba-1 (Wako, Richmond, VA 019-19,741, 1:1000, O/N, RT), glial fibrillary acidic protein (GFAP) (Dako, Cambridge, United Kingdom Z0334, 1:4000, O/N, RT), APC CC1 (Calbiochem OP-80, 1:100, O/N, RT).

### 2.7. Imaging and quantification

DAB stains were imaged using a Nikon Coolpix E4500 digital camera attached to a Nikon Eclipse E800 microscope. Images of fluorescent stains were captured using a Hamamatsu CCD C4742 digital camera attached to a Leica DMR microscope using Volocity software (PerkinElmer, Waltham, MA). To study colocalisation, images were captured using a Leica TCS SPE Laser scanning confocal microscope. A single focal plane was used for any colocalisation image presented using confocal microscopy.

Immunopositive cells for all sections were counted manually with the experimenter blind to treatment. The immunopositive cells were separated by laminae I–II and III–V. Once all sections were counted, per animal, 5 sections with the maximum response (ipsilateral) were used and the average taken on the ipsilateral and, when relevant, contralateral side of the dorsal horn. The average of the treatment group was then calculated along with the SE of each group mean (SEM).

If the goal was to obtain a percentage overlap of 2 labelled populations, cells of the first population were quantified using the above protocol. Then, in the same tissue sections, the second population of interest, as well as the population of double-labelled cells, was quantified. The percentage of double labelling was then calculated for each section of tissue, and averaged per animal. Finally, the average percentage of double labelling for the group was presented as mean ± SEM. Because the maximal expression of 2 populations of interest may not occur in the same tissue sections, out of interest, the maximum percentage overlap was also calculated by selecting the 5 tissue sections with maximal response from the second population. However, there was never any difference in percentage overlap values when using 5 sections with maximum response per animal from either combination of stain.

### 2.8. Statistical analysis

All statistical analyses were performed using IBM SPSS PC+ (Chicago, IL) or Microsoft Excel (Redmond, WA). Analyses were performed using independent *t* tests or 2- or 3- way, if relevant repeated-measures, analysis of variance (ANOVA), as appropriate, followed by univariate post hoc analysis. A significant main effect of treatment or significant interaction was a condition to follow on with post hoc analysis. Data were checked for normal distribution using the Shapiro–Wilk analysis, and homogeneity of variance using the Levene test before performing parametric analyses. Data were checked for sphericity using the Mauchly test of sphericity and when violated, the Greenhouse–Geisser correction was used. For all experiments, a statistical significance level of *P* < 0.05 was used.

## 3. Results

### 3.1. p-H3S10 is upregulated in ipsilateral dorsal horn neurons of the spinal cord after noxious hind paw stimulation by formalin and capsaicin

**Figure 1.**
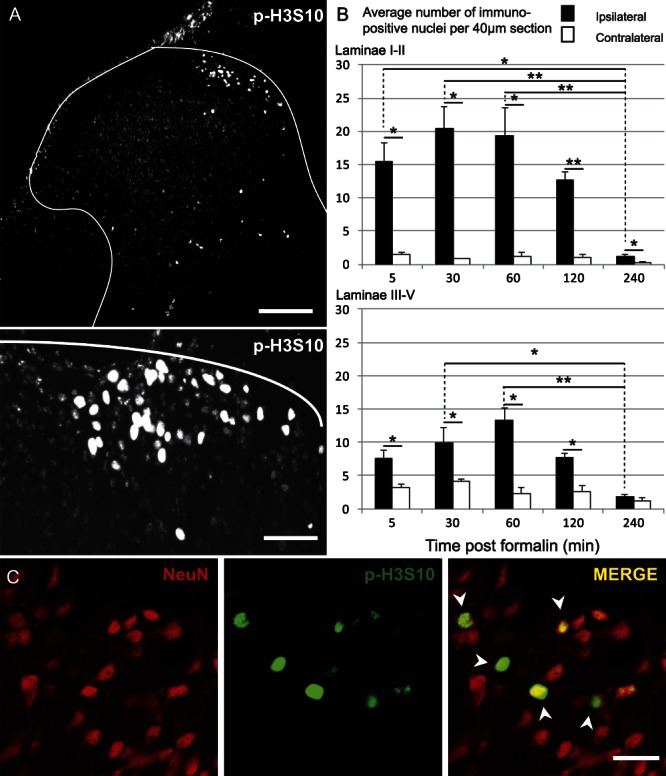
p-H3S10 rapidly increases in the ipsilateral dorsal horn after inflammation was induced through hind paw formalin injection and is expressed predominantly in neurons. (A) Representative images of nuclear p-H3S10 distribution within the L4 dorsal horn 30 minutes after formalin injection. White lines indicate border between gray and white matter. Low magnification: scale bar, 50 μm. High magnification: scale bar, 30 μm. (B) Ipsilateral and contralateral counts of p-H3S10 in laminae I and II and laminae III–V (n = 3 each time point, 5 sections per animal). Data show mean ± SEM (per 40 μm section). *P < 0.05; **P < 0.01; Bonferroni post hoc analysis for comparisons across ipsi sides and paired *t* test for comparison ipsi vs contra. (C) p-H3S10 (green) and NeuN (red) expression in formalin-stimulated animals. In the merge, yellow indicates colocalisation. Arrowheads indicate examples of colocalisation. Scale bar, 25 μm.

There was a rapid increase in p-H3S10-labeled nuclei in the medial superficial dorsal horn as early as 5 minutes after intraplantar formalin injection (Fig. 1A, B). The number of p-H3S10-labeled nuclei increased only on the ipsilateral side (2-way ANOVA: differences across time points: F_(4,10)_ = 8.5, *P* < 0.01, post hoc analysis for the ipsi side only: F_(4,10)_ = 7.8, *P* < 0.01; ipsi vs contra: F_(1,10)_ = 104.2, *P* < 0.0001). Expression of p-H3S10 in superficial laminae I–II was maximal at the 30-minute time point (mean, 20.5 ± 3.2 cells per 40-μm section). In deeper laminae III–V, maximum expression of p-H3S10 occurred at 1 hour (mean, 13.3 ± 1.7 cells per 40-μm section; 2-way ANOVA: differences across time points: F_(4,10)_ = 9.9, *P* < 0.01, post hoc analysis only for the ipsi side: F_(4,10)_ = 8.3, *P* < 0.01; ipsi vs contra: F_(1,10)_ = 49.4, *P* < 0.0001). By 4 hours, p-H3S10 levels were back to baseline. We also observed the expression of p-H3S10 in the ipsilateral superficial dorsal horn at 1 hour after intraplantar capsaicin stimulation (10.0 ± 1.0 nuclei across lamina I–II per 40-μm section, n = 4). Previous studies have shown that p-H3S10 occurs in neurons.^18^ To identify whether formalin-induced spinal p-H3S10 was also occurring in neurons, we performed colocalisation of p-H3S10 and the neuronal marker NeuN. We found that formalin-induced p-H3S10 nuclei colocalised almost exclusively with NeuN in the dorsal horn (Fig. 1C). p-H3S10 was not seen to colocalise with glial cells using the astrocyte marker, GFAP, the oligodendrocyte precursor cells marker APC CC1, or Iba-1, a microglia marker (Figure S1 available online as Supplemental Digital Content at http://links.lww.com/PAIN/A323).

Thus, p-H3S10 was rapidly and transiently upregulated in neurons of the superficial dorsal horn after peripheral noxious stimulation. We next characterised the neuronal population expressing p-H3S10.

### 3.2. p-H3S10 upregulation occurs in neurons of the pain pathways

p-ERK, c-Fos, and Zif268 have been shown to be rapidly and transiently upregulated after peripheral stimulation.^31,32,53^ We therefore investigated whether p-H3S10 could be coexpressed with these markers. Moreover, NK1 receptor (NK1R)-positive neurons are important for nociceptive regulation,^43^ and we also looked at the coexpression of p-H3S10 and NK1R.

#### 3.2.1. p-H3S10 is expressed in p-ERK-positive neurons

**Figure 2.**
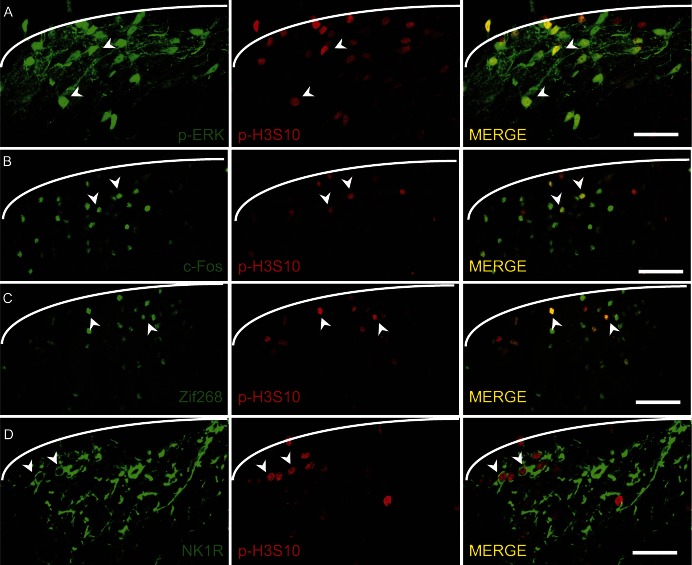
p-H3S10 expression occurs within neurons of the pain pathways. (A) Dorsal horn images of p-ERK (green) and p-H3S10 (red) double labelling in laminae I and II. In the merge, colocalisation is seen in yellow. Scale bar, 30 μm. (B) Dorsal horn images of c-Fos (green) and p-H3S10 (red) double labeling in formalin-stimulated animals. In the merge, colocalisation is seen in yellow. Scale bar, 50 μm. (C) Dorsal horn images of Zif268 (green) and p-H3S10 (red) double labeling. In the merge, colocalisation is seen in yellow. Scale bar, 50 μm. (A-D) White lines indicate medial border between lamina I and white matter; arrowheads indicate examples of colocalisation. All sections are 40 μm shown taken in a single focal plane in a formalin-stimulated animal. (D) Dorsal horn images of NK1R (green) and p-H3S10 (red) double labeling. The final image shows a merge of the first 2 images; NK1R is a cell-surface receptor and thus colocalisation is shown as NK1R surrounding the p-H3S10-labelled nucleus. Scale bar, 30 μm.

We first investigated the colocalisation of p-H3S10 nuclei and p-ERK. Maximal expression of p-ERK occurs 5 minutes after formalin injection,^32^ and levels remain elevated for at least 30 minutes. This allowed us to assess the coexpression of p-H3S10 and p-ERK at this time point. In laminae I–II, 51% ± 7% of all p-H3S10-expressing cells colabelled with p-ERK, whereas 44% ± 3% of p-ERK-expressing cells also expressed p-H3S10 (mean p-ERK population 30.7 ± 7.4 cells per 40-μm section; mean number of double-labelled cells: 13.9 ± 3.8 per 40 μm section) (Fig. 2A). In laminae III–V, similar percentages of colocalisation were observed: 49% ± 8% of p-H3S10-expressing cells coexpressed p-ERK and 35% ± 6% of p-ERK-expressing cells also coexpressed p-H3S10 (mean p-ERK population 16.6 ± 5.5 cells per 40-μm section; mean number of double-labelled cells: 5.1 ± 1.1 per 40-μm section).

#### 3.2.2. p-H3S10 coexpression with immediate early gene products c-Fos and Zif268

The immediate early gene (IEG) products c-Fos and Zif268 are markers of neuronal plasticity known to have maximum expression in the ipsilateral dorsal horn 1 to 2 hours after noxious stimulation. Robust upregulation of both c-Fos and Zif268 was observed 1 hour after formalin injection. Both populations had a larger and more anatomically widespread distribution compared with that of the p-H3S10 population (Fig. 2B, C). In lamina I–II, 61% ± 8% of p-H3S10-positive neurons coexpressed c-Fos, whereas 17% ± 3% of c-Fos-positive neurons coexpressed p-H3S10 (mean c-Fos population 35.2 ± 2.3 cells per 40-μm section; mean number of double-labelled cells: 6.2 ± 1.2 per 40 μm section). Of note, 57% ± 5% of the p-H3S10 population of neurons coexpressed Zif268, whereas 12% ± 1% of the Zif268 population coexpressed p-H3S10 (mean Zif268 population 53.6 ± 7.7 cells per 40-μm section; mean number of double-labelled cells: 6.3 ± 1.2 per 40-μm section).

#### 3.2.3. p-H3S10 is expressed in NK1R-positive neurons

Excitatory NK1R-positive neurons are key to nociceptive processing. Indeed, selective ablation of these neurons was shown to significantly reduce injury-induced hypersensitivity.^43^ Analysis of p-H3S10 colocalisation with NK1R-positive neurons at 1 hour after formalin injection indicated that 4.5% ± 2% of laminae I and II p-H3S10 coexpressed NK1R and in laminae III–V 4% ± 1% of p-H3S10 colabelled NK1R (Fig. 2D). These numbers were expected considering the small NK1 population size. We also calculated that 17% ± 8% of lamina I NK1R-expressing neurons coexpressed p-H3S10 (mean NK1R population: 6.5 ± 1.5 cells per 40-μm section; mean number of double-labelled cells: 1.3 ± 0.7 per 40-μm section) and in laminae III–V, 15% ± 6% of NK1R-positive cells coexpressed p-H3S10 (mean NK1R population 6.1 ± 1.2 cells per 40 μm section; mean number of double-labelled cells: 0.8 ± 0.4 per 40 μm section).

Our results showed that formalin-induced p-H3S10 nuclei colocalised with p-ERK, c-Fos, Zif268, and NK1R. This suggested that p-H3S10 could play an important role in nociception.

### 3.3. Depletion of spinal serotonin (5-HT) prevents the full expression of formalin-induced p-H3S10

Nociceptive signalling in the superficial dorsal horn is known to be regulated by descending controls from the brain. We therefore investigated whether p-H3S10 expression was modulated by descending serotonergic circuits that are known to regulate dorsal horn activity.^44^

**Figure 3.**
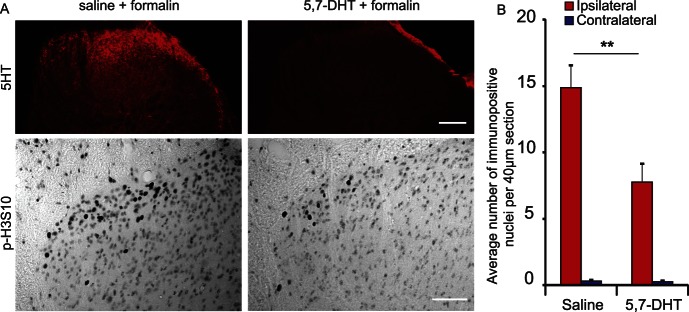
Depletion of spinal serotonin prevents the full expression of formalin-induced p-H3S10. (A) Typical dorsal horn image of 5-HT and p-H3S10 staining in animals receiving i.t. saline or i.t. (5,7-DHT), 30 minutes after formalin stimulation. Scale bar, 50 μm (upper), 30 μm (lower). (B) Counts of p-H3S10 nuclei in the ipsilateral and contralateral dorsal horn (n = 7 each group, 5 sections per animal). Data show group mean ± SEM (per 40-μm section). **P < 0.01.

We used i.t. delivery of the neurotoxin 5,7-DHT to lesion 5-HT-containing descending fibres from the RVM and found that 5,7-DHT caused a specific depletion of 5-HT in the spinal cord (Fig. 3A) and a 50% reduction in p-H3S10 nuclei 30 minutes after formalin injection compared with the vehicle (2-way ANOVA, factor treatment F_(1,24)_ = 9.44, *P* < 0.01, side x treatment interaction: F_(1,24)_ = 9.14, *P* < 0.01) (Fig. 3B). There were no indication of neuronal death or gliosis as shown by NeuN or GFAP immunostaining at 30 minutes after formalin injection in animals depleted of spinal 5-HT (Figure S2, available online as Supplemental Digital Content at http://links.lww.com/PAIN/A323).

The RVM is known to have bidirectional inhibitory and facilitatory descending influences on spinal nociception.^52^ However, the majority of studies using 5,7-DHT to lesion 5-HT-containing fibres have concluded an overall facilitatory role for 5-HT on spinal nociception.^24,50^ Our findings here, showing that 5-HT is required for the full expression of formalin-induced p-H3S10, would therefore support a pronociceptive role for p-H3S10. To characterise further the potential role of spinal histone H3 phosphorylation at S10 in nociception, we next investigated the molecular signalling events involved in formalin-induced p-H3S10 expression.

### 3.4. The majority of formalin-induced p-H3S10 nuclei are downstream of ERK signalling and colocalise with p-MSK1

**Figure 4.**
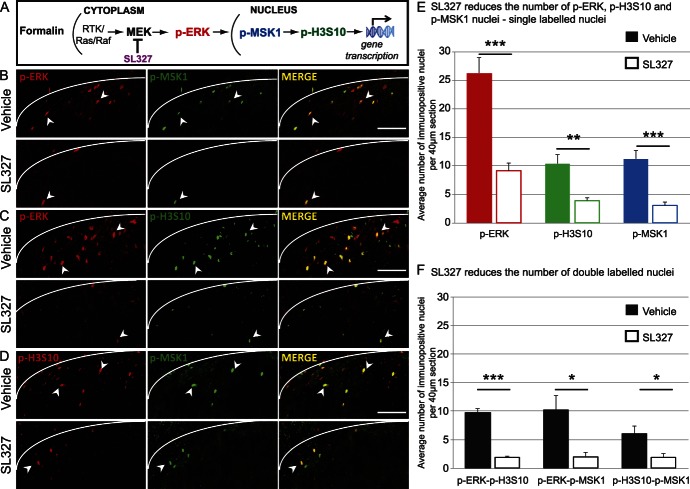
Spinal extracellular signal-regulated kinase (ERK) regulates p-MSK1 and p-H3S10 expression after formalin injection. (A) Diagram of postulated signaling pathways upstream of p-H3S10. (B) Dorsal horn images of p-ERK and p-MSK1 in vehicle- and MEK inhibitor SL327-treated animals 30 minutes after formalin stimulation. (C) Dorsal horn images of p-ERK and p-H3S10 in vehicle- and MEK inhibitor SL327-treated animals 30 minutes after formalin stimulation. (D) Dorsal horn images of p-H3S10 and p-MSK1 in vehicle- and MEK inhibitor SL327-treated animals 30 minutes after formalin stimulation. (B-D): Pictures show a single focal plane. Final column images show a merge of the first 2 images; colocalisation is seen in yellow; arrowheads indicate examples of colocalisation. The white line indicates the medial border between lamina I and white matter. Scale bar, 50 μm. (E) Quantification of p-ERK, p-H3S10, and p-MSK1 single-labeled cells 30 minutes after formalin injection. n = 8 in each treatment group, 5 sections per animal. (F) Quantification of p-ERK, p-H3S10, and p-MSK1 double-labeled cells 30 minutes after formalin. n = 4 in each group. (E-F) Values presented as group mean ± SEM (per 40 μm section). *P < 0.05, **P < 0.01, ***P < 0.001, SL327-treated vs vehicle-treated animals.

Our data so far suggested that p-H3S10 was partially dependent on ERK/MAPK signalling. The role of MEK, the upstream kinase for p-ERK, in nocifensive formalin behaviours has already been demonstrated.^33^ In particular, the MEK inhibitor SL327 has been shown to attenuate both the first and second phase of the formalin response.^1^ p-MSK1 is a well-characterised kinase directly downstream of p-ERK which can induce p-H3S10^49^ (Fig. 4A). However, the role of p-MSK1 in pain processing has never been investigated. In this study, SL327 was used as a pharmacological tool to investigate the expression of molecular targets downstream of ERK, specifically p-MSK1 and p-H3S10, after noxious stimulation.

Formalin stimulation induced expression of p-ERK, p-H3S10, and p-MSK1 in laminae I and II of the ipsilateral dorsal horn at 30 minutes (26.3 ± 2.7, 10.4 ± 1.5, and 11.2 ± 1.5 cells per 40-μm section, respectively) (Fig. 4B, C, D and E). Crucially, the great majority (87% ± 3%) of p-MSK1 nuclei colocalised with p-ERK (Fig. 4B, F) and 85% ± 5% of p-H3S10 nuclei coexpressed p-MSK1 (with 59% ± 5% of the p-MSK1 population coexpressing p-H3S10; Fig. 4D, F).

There was a significant effect of SL327 on the expression of p-ERK, p-MSK1, and p-H3S10 in laminae I and II of the ipsilateral dorsal horn (2-way ANOVA: F_(1,42)_ = 69.4; *P* < 0.001; Fig. 4B, C, D, and E). After administration of systemic SL327, there was a 65% reduction of p-ERK (F_(1,14)_ = 31.9, *P* < 0.0001) (Fig. 4E). SL327 also caused a significant reduction (73%) in total p-MSK1 nuclei (F_(1,14)_ = 23.9, *P* < 0.0001) (Fig. 4E), and the majority of the remaining p-MSK1-positive nuclei (63% ± 14%) still colocalised with p-ERK. There was also a significant reduction in double-labelled cells (p-ERK + p-MSK1; Fig. 4F; F_(1,6)_ = 12.2; *P* < 0.05). After SL327 administration, we also found a 62.5% reduction in total p-H3S10 nuclei in laminae I and II of the ipsilateral dorsal horn (F_(1,14)_ = 16.4, *P* < 0.001) (Fig. 4E), and 46% ± 8% of the remaining p-H3S10 population still colocalised with p-ERK after SL327 administration (Fig. 4F). Again, there was a significant reduction in double-labelled cells after SL327 administration: p-ERK+ p-H3S10; F_(1,6)_ = 138.2; *P* < 0.0001 and p-MSK1 + p-H3S10; F_(1,6)_ = 11.3; *P* < 0.05 (Fig. 4F).

All together, these results strongly suggested that the expression of p-MSK1 and p-H3S10 in the superficial dorsal horn was mainly downstream of ERK signalling. We next investigated whether p-H3S10 was downstream of MSK1 signalling and whether MSK1 activity might contribute to nociceptive processing.

### 3.5. Inhibition of MSK1 with SB727651A reduces formalin-induced p-H3S10 expression and prevents the full expression of formalin-induced nocifensive behaviour

The previous experiments indicated that the majority the p-H3S10 population colabelled with the p-MSK1 population (85%) and that p-MSK1 and p-H3S10 populations were of similar size. MSK1 therefore seemed to be an ideal pharmacological target to manipulate p-H3S10 expression.

#### 3.5.1. Inhibition of MSK1 with SB747651A reduces formalin-induced p-H3S10 expression

**Figure 5.**
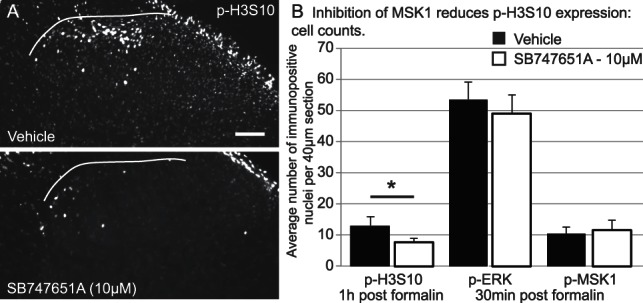
Inhibition of MSK1 with SB747651A (10 μM) causes a reduction in formalin-induced p-H3S10 but has no effect on p-ERK and p-MSK1 expression. (A) Images of spinal p-H3S10 in vehicle- and SB747651A (10 μM)-treated animals, 1 hour after formalin injection. Scale bar, 100 μm; the white line indicates the medial border of lamina I and white matter. (B) Quantification of p-H3S10, p-ERK, and p-MSK1 in the dorsal horn of the vehicle or SB747651A (10 μM) formalin-treated animals (n = 8 in each group, 5 sections per animal). Values presented as group mean ± SEM (per 40 μm section).

The MSK1 inhibitor, SB747651A, was delivered i.t. (1 or 10 μM, delivered in 10 μL) 30 minutes before formalin stimulation. There was no effect of i.t. SB747651A (1 μM) on spinal p-H3S10 expression, but treatment with i.t. SB747651A (10 μM dose) led to a significant reduction (42%; *P* < 0.05, independent Student *t* test) in spinal p-H3S10 1 hour after formalin injection (vehicle-treated animals: 13.1 ± 2.8 cells per 40-μm section; SB727651A-1 μM: 9.5 ± 1.4 cells; and SB727651A-10 μM: 7.6 ± 1.3 cells) (Fig. 5A, B). Control experiments showed that there was no change in the expression of upstream p-ERK and p-MSK1 between the vehicle and SB747651A (10 μM dose) groups 30 minutes after formalin injection (Fig. 5B).

#### 3.5.2. SB747651A prevents the full expression of formalin-induced nocifensive behaviour

**Figure 6.**
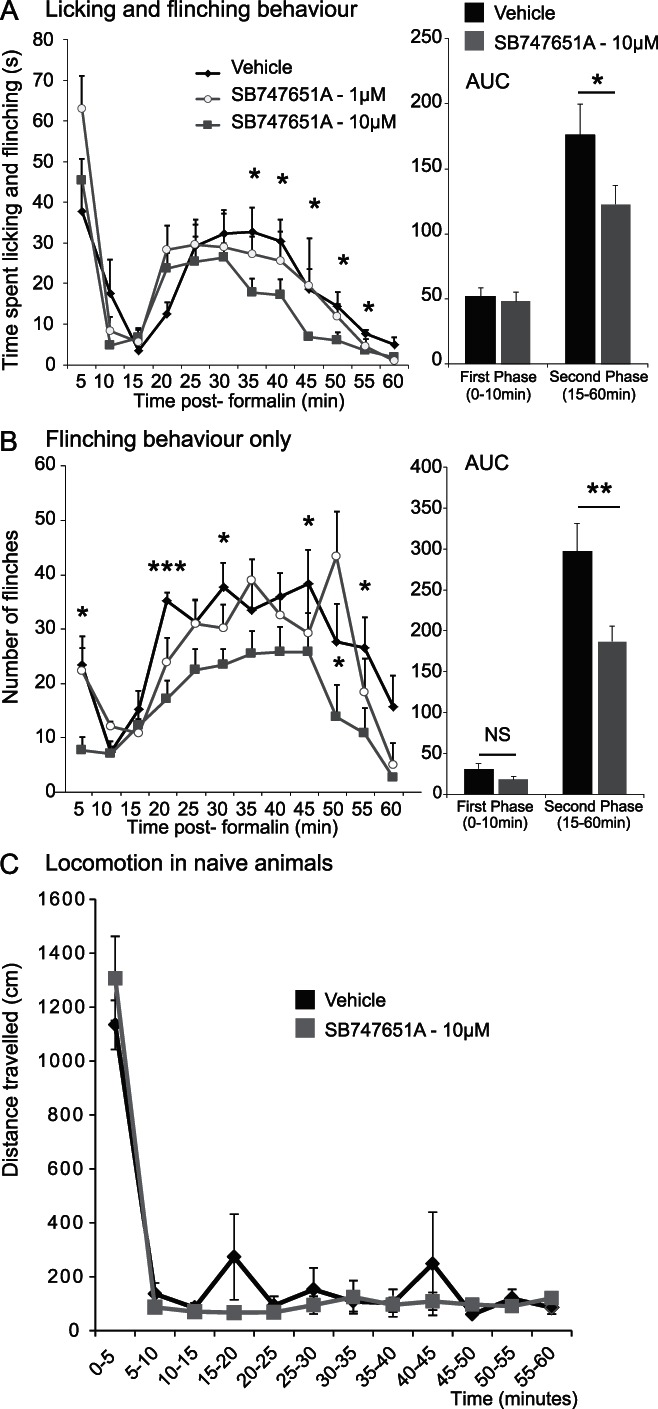
SB747651A (10 μM) attenuates nocifensive behaviour but does not impair motor function. (A) SB747651A (10 μM) attenuated the time spent licking and flinching during the second phase of the formalin response (35-60 minutes after formalin injection). AUC comparison indicated a 30% reduction of time spent licking/flinching in SB747651A (10 μM)-treated animals during the second phase (15-60 minutes) of the formalin response. N = 11 to 16. (B) SB747651A (10 μM) also reduced the formalin-induced number of flinches during the first and second phases of the formalin response. AUC comparison indicated a 43% reduction of flinch behaviour during the first phase (0-10 minutes) and 37% reduction during the second phase (15-60 minutes) in SB747651A (10 μM)-treated animals. N = 8 to 10. (C) There were no differences in the total distance travelled in the open-field paradigm between vehicle- and SB747651A (10 μM)-treated naive animals. (n = 3/4 in each group). (A-C). Mean ± SE mean. *P < 0.05, **P < 0.01; ***P < 0.001 between vehicle and 10 μM SB747651A.

We finally investigated the effects of SB747651A on nocifensive behaviour. Formalin response behaviours were scored for 1 hour after stimulation with the vehicle, 1 or 10 μM SB747651A. As above, the 1 μM had no effect on formalin-induced nocifensive behaviour (Fig. 6A). However, 10 μM SB747651A attenuated the formalin response compared with the vehicle-treated group. There was a significant main effect of treatment between 35 and 55 minutes on the time spent licking and flinching (2-way repeated-measure ANOVA, F_(2,30)_ 5.7, *P* < 0.01; post hoc analysis vehicle vs 10 μM: F_(1,25)_ = 10.9, *P* < 0.01; Fig. 6A). Calculation of area under the curve (AUC) indicated a 30% reduction of time spent licking/flinching in 10 μM SB747651A-treated animals during the second phase (15–60 minutes) of the formalin response (*P* < 0.05, independent Student *t* test) (Fig. 6A).

Interestingly, SB747651A significantly attenuated both the first and second phase of the formalin response when quantified by the total number of flinches alone (2-way repeated-measure ANOVA, time 5-10 minutes, time × treatment interactions: F_(2,21)_ = 5.1, *P* < 0.05; post hoc analysis vehicle vs 10 μM: time × treatment interactions F_(1,16)_ = 9.2, *P* < 0.01; time 15-60 minutes, treatment: F_(2,21)_ = 3.8, *P* < 0.05; post hoc analysis vehicle vs 10 μM: F_(1,16)_ = 7.6, *P* < 0.05, Fig. 6B). AUC calculation indicated a significant 37% reduction during the second phase (15-60 minutes) in SB747651A-treated animals (*P* < 0.01, independent Student *t* test). AUC for the first phase formalin was however not significant as it combined 2 time bins, 5 and 10 minutes (Fig. 6B). Finally, the 10-μM dose of SB747651A had no effect on locomotion in naive animals, as measured in the open-field paradigm (Fig. 6C).

## 4. Discussion

It is widely known that p-ERK is a marker of neural activation after noxious stimulation and that spinal ERK regulates nocifensive behaviour. Less is known, however, about the molecular signalling events downstream of p-ERK that lead to gene regulation after injury. Extracellular signal-regulated kinase and MSK1 regulate p-H3S10 during memory formation,^13,14^ a process that requires cellular activity similar to that seen in sensitised states after injury.^34^ In this study, we have shown for the first time that activation of spinal MSK1 regulates the molecular events downstream of ERK, including the phosphorylation of histone H3 at serine 10, and formalin-induced nocifensive behaviour.

### 4.1. Extracellular signal-regulated kinase regulation of p-H3S10 in models of synaptic plasticity

p-ERK is a classical marker of primary afferent nociceptive activation and central sensitisation.^23^ p-H3S10 is known to occur downstream of ERK through MSK1,^49^ and strong coupling of p-ERK and p-H3S10 has been confirmed in various models of activity-induced synaptic plasticity in the brain.^7,14,16,18^ One hour after pharmacological activation of neuronal activity with kainic acid, 80% of p-H3S10 nuclei colocalised with p-ERK in neurons of the dentate gyrus.^18^ Furthermore, p-H3S10 was found to be highly coexpressed (94%) with p-ERK in the lateral septum of the brain 1 hour after plasticity induced by naloxone-precipitated morphine withdrawal.^15^ Our results, showing that more than half of the spinal p-H3S10 population overlapped with p-ERK 30 minutes after formalin, suggested that p-H3S10 could also be downstream of p-ERK signalling in the superficial dorsal horn.

Pharmacological blockade of ERK signalling with the MEK inhibitor SL327 was a crucial experiment to investigate the contribution of ERK signalling on p-MSK1 and p-H3S10 expression. The reduction in both formalin-induced p-MSK1 and p-H3S10 after SL327, by 73% and 62.5%, respectively, indicated a strong requirement of ERK signalling for the full expression of p-MSK1 and p-H3S10, as previously shown.^7,9,10,14,15,45^ It is important to note that p-ERK, p-MSK1, and p-H3S10 peak at different time points, and therefore the percentages of exact overlap are most probably greater than those suggested here. To conclusively establish that p-H3S10 is entirely downstream of p-ERK would require the complete elimination of p-ERK—a condition that was not fulfilled with 50 mg/kg SL327, a dose previously used by others.^1^ Therefore, whether an ERK-independent signalling cascade could induce spinal p-H3S10 remains to be explored.

### 4.2. Targeting p-H3S10 through MSK1

MSK1 was an ideal target for interfering with p-H3S10 because it is upstream of p-H3S10 and 85% of S10 nuclei also expressed p-MSK1. It should be noted that another kinase, p70 ribosomal s6 kinase (RSK2), that lies downstream of ERK, might also activate p-H3S10.^16^ Moreover, MSK1 had never been explored within the context of nociception, despite being a direct downstream target of ERK. The induction of p-MSK1 observed at 30 minutes after stimulation was in agreement with a previous study that showed neuronal induction of p-MSK1 after 15 to 30 minutes of glutamate stimulation in vitro.^6^

Many studies have used genetically modified mice to show that MSK1 regulates neural plasticity, in particular memory formation.^8,9,13,17,35^ MSK1 has also been shown to modulate dendritic morphology and synaptic plasticity involved in hippocampal environmental enrichment and homeostatic synaptic scaling. This is believed to happen by CREB-mediated regulation of AMPA subunit GluR1 expression, through the IEG arc/arg3.1, which is upregulated in the spinal dorsal horn by noxious stimulation.^17,29^

Recently, a new inhibitor with improved selectivity for MSK1, SB747651A, has become available (Tocris, Bristol, United Kingdom).^42^ There were no upstream changes in ERK activation after administration of SB747651A, supporting the targeted specificity of MSK1 inhibition. There were also no changes in p-MSK1 expression. This was expected as SB747651A is a specific inhibitor of the MSK1 N-terminal kinase, the domain reportedly essential for the phosphorylation of downstream MSK1 substrates.^40^ The antibody used to detect p-MSK1 was specific to phosphorylation at threonine 581 in the C-terminal kinase domain, activity that precedes N-terminal activity. SB747651A reduced formalin-induced p-H3S10 by 50%, confirming that p-H3S10 was downstream of p-MSK1 signalling. SB747651A also reduced formalin-induced nocifensive behaviour, revealing for the first time the role of MSK1 in nociceptive processing.

### 4.3. Inhibition of MSK1 reduces formalin-induced nocifensive behaviour

Many studies have investigated the effect of ERK inhibition on formalin-induced nocifensive behaviour. Extracellular signal-regulated kinase is often targeted by inhibition of its upstream kinase, MEK, using inhibitors such as PD98059 and U0126. Regardless of the behaviour observed (ie, time licking/lifting or number of flinches), all studies have reported a reduction of nocifensive behaviour after MEK inhibition in the second phase of the formalin test.^32,38,50,55^ Although the extent of the inhibition differs widely across studies, a 30% to 80% decrease is usually reported. In this study, we found that MSK1 inhibition with SB747651A inhibited nocifensive behaviours in the second phase of the test by 30% to 50%, which would suggest that MSK1 signalling is one of the main signalling pathways downstream of ERK that contributes to sensitised states.

We also observed a reduction in the number of flinches in the first phase of the formalin test, and it has been argued that, in rats, flinches could be a better reflection of nociceptive responses than licking.^4,41^ This would suggest that SB747651A indeed reduces nociceptive behaviour in the first phase of the formalin response. It should be noted that the reduction in flinching, measured in numbers, could not be seen when time spent licking and flinching was plotted because the time spent licking was much greater than the time spent flinching. Moreover, although there are no reports of a reduction of the nocifensive behaviours in the first phase of the formalin test, most studies using MEK inhibitors show a trend for a decrease at a specific early time point, and this trend is often lost when the first phase is analysed as a longer time bin, as seen in our study. Finally, it should be mentioned that although we ascribed the molecular and behavioural effects of SB747651A and the MEK inhibitor SL327 to the modulation of the ERK and MSK1 signalling pathways, there is a possibility that these observations could also be partially attributed to off-target effects.

### 4.4. p-H3S10 regulation of specific gene programmes

Evidence from previous studies of learning and memory suggest that ERK-dependent MSK1 actively contributes to the regulation of gene expression through direct manipulation of p-H3S10. Because of the partial overlap between p-H3S10 and other nociceptive markers, such as c-Fos and Zif268, it is highly likely that the consequences of histone H3 phosphorylation at S10 impact on a number of gene programs in various subsets of cells. Although the downstream targets through which p-ERK contributes to sensitised states remain largely unknown, the results of our study suggest that this could occur through upregulation of the CREB-GluR1 pathway that has been shown to be modulated by MSK1.^17,29^ Moreover, a number of genes under the regulation of the transcription factor CREB could also be under the regulation of p-H3S10. As the general function of p-H3S10 is to aid in the relaxation of chromatin, it is possible that p-H3S10 and CREB (both through phosphorylation by MSK1) could act together to permit transcription.

Candidate genes under the regulation of p-H3S10 activity are likely to be plasticity related and, indeed, p-H3S10 has been shown to be directly responsible for the induction of the IEGs c-Fos and Zif268 in granule neurons of the dentate gyrus.^27^ Virtually all of p-H3S10 neuronal nuclei have been shown to coexpress c-Fos mRNA, with PH3S010 expression always preceding that of c-Fos in the dentate gyrus.^18^ Our data indicated that at least 61% of p-H3S10 expressed c-Fos 1 hour after formalin injection and that the percentage overlap of p-H3S10/p-ERK occurred at around 50%. This suggested that at least 10% of the p-H3S10 population expressed both p-ERK and c-Fos, indicating a potential ERK-dependent regulation of c-Fos through p-H3S10. Others have shown that spinal ERK has significant regulation over c-Fos expression after nociceptive activation^36^ and that ERK controlled c-Fos expression in a p-MSK1- and p-H3S10-dependent mechanism in models of addiction.^7,46^ However, the fact that in our study only 17% of c-Fos-positive neurons coexpressed p-H3S10 also suggests that other routes contribute to c-Fos activation. The IEG *Zif268* is also known to regulate nociceptive processing,^47^ and our results indicated that 57% of p-H3S10 expressed Zif268. Experiments using ChIP have shown that upregulation of Zif268 occurs in the brain under the regulation of p-H3S10 during cognitive processing,^26^ and ERK regulation of Zif268 has been well established in striatal and dentate gyrus neurons exhibiting LTP.^19,48^ However, less is known about ERK regulation of Zif268 in the spinal cord.

In conclusion, our study is the first to elucidate the phosphorylation of MSK1 in the superficial dorsal horn as a molecular event downstream of ERK signalling that contributes to the full expression of pain states. Moreover, we report that dorsal horn neurons are rapidly epigenetically tagged with p-H3S10 after peripheral noxious stimulation and that this occurs downstream of MSK1 and ERK signalling. These results strongly suggest that p-H3S10 could regulate spinal nocispecific gene programmes after peripheral injury.

## Conflict of interest statement

The authors have no conflicts of interest to declare.

This work was supported by the MRC Grants G1100577 and G0801381.

## Supplementary Material

SUPPLEMENTARY MATERIAL

## Appendix A Supplemental Digital Content

Supplemental Digital Content associated with this article can be found online at http://links.lww.com/PAIN/A323.
